# Visual saliency guided perceptual adaptive quantization based on HEVC intra-coding for planetary images

**DOI:** 10.1371/journal.pone.0263729

**Published:** 2022-02-09

**Authors:** Yuqi Dai, Changbin Xue, Li Zhou

**Affiliations:** 1 National Space Science Center, Chinese Academy of Sciences, Beijing, China; 2 University of Chinese Academy of Sciences, Beijing, China; Nanjing University of Information Science and Technology, CHINA

## Abstract

Due to the limited storage space of spacecraft and downlink bandwidth in the data delivery during planetary exploration, an efficient way for image compression onboard is essential to reduce the volume of acquired data. Applicable for planetary images, this study proposes a perceptual adaptive quantization technique based on Convolutional Neural Network (CNN) and High Efficiency Video Coding (HEVC). This technique is used for bitrate reduction while maintaining the subjective visual quality. The proposed algorithm adaptively determines the Coding Tree Unit (CTU) level Quantization Parameter (QP) values in HEVC intra-coding using the high-level features extracted by CNN. A modified model based on the residual network is exploited to extract the saliency map for a given image automatically. Furthermore, based on the saliency map, a CTU level QP adjustment technique combining global saliency contrast and local saliency perception is exploited to realize a flexible and adaptive bit allocation. Several quantitative performance metrics that efficiently correlate with human perception are used for evaluating image quality. The experimental results reveal that the proposed algorithm achieves better visual quality along with a maximum of 7.17% reduction in the bitrate as compared to the standard HEVC coding.

## 1. Introduction

The image data collected during the planetary exploration has significant research value for scientists to analyze the special geographical and geological environment. The volume of the original high-resolution image is large and redundant, which imposes an urgent demand for efficient and reliable image compression techniques, considering the performance limitations of spacecraft equipment and the complex deep space communication environment. Consequently, image compression onboard is crucial in saving the transmission bandwidth and reducing data transmission time [1].

Image compression is applied to reduce the volume of data by removing redundant information in the spatial and time domains, along with other statistical aspects. For most space missions, given the data values and high cost of space missions, lossless compression methods including prediction-based techniques such as DPCM (Differential Pulse Code Modulation) [2], lossless JPEG [3] and JPEG-LS [4] and transform-based methods such as JPEG-2000 [5] and CCSDS-IDC [6] have been commonly used. However, lossless compression can only achieve a significantly low compression ratio than lossy compression techniques, typically ranging from about 1.5:1 to 3:1 [7]. Considering most deep space exploration missions like Mars rovers and spacecrafts, have long-term goals, the number of cameras carried on is more than one and every camera has its own task and priorities [8]. Moreover, the compression requirements of image data captured by the same camera vary in different scenarios of the same task. Therefore, the research of lossy image compression methods to provide flexible and powerful compression ratio control capabilities also has great application significance.

Over the past couple of decades, image compression techniques have been developing rapidly, and the hybrid compression framework formed by prediction, transformation, quantization, and entropy coding has become preferable. Several new module optimization methods are being proposed. Meanwhile, researchers focusing on content-aware encoding attempt to improve the coding efficiency by removing visual redundancy to further improve the compression ratio. One notable approach is to use saliency detection for region-wise quantization control before the traditional image compression framework [9].

The visual saliency-based coding optimization method can be applied to any image compression system because it does not need any modification in the syntactic structure of bitstream. Akbari et al. [10] proposed a saliency-driven image compression method based on adaptive sparse representations. In this method, the rate allocation was computed according to a saliency detection model based on graph theory. Ku et al. [11] proposed a novel bit allocation scheme for HEVC [12] based on saliency fusion weights composed of the low-level feature and inter-frame correlation feature. Mahalingaiah et al. [13] modified the quantization quality parameters according to the saliency information and gained better visual quality compared to the standard JPEG encoder. Zhu et al. [14] proposed a spatiotemporal visual saliency guided perceptual HEVC method, wherein the spatial saliency was extracted by CNN, and temporal saliency was computed from the compressed-domain motion information. Based on the spatial-temporal joint saliency feature, the range of the quantization parameter was dynamically adjusted. In [15], Chiang et al. proposed a saliency prediction model based on the stereographic projection method for 360-degree images. According to the saliency map, this model optimized the global rate-distortion to improve the image quality of Region-of-Interest (ROI) while reducing the overall bitrate. The authors in [16] combined the video encoder with a deep saliency detection model for quantization bit allocation and reduced the code rate by 17% under the same coded image quality condition. To improve the coding quality of salient regions for each frame, Sun et al. [17] proposed an adaptive rate control method based on the fusion saliency map consisting of static and dynamic salient feature computed from the deep CNN model and motion target segmentation algorithms.

The aforementioned techniques are deployed ahead of image compression for guiding the encoder to remove psycho-visual redundancy and improve the visual quality of images while simultaneously reducing the bitrate. Accordingly, the complexity of the whole pipeline increases significantly. Hence, an efficient saliency region extraction algorithm and reasonable strategy for coding resource allocation are the key parameters to improve the coding efficiency.

**Fig 1:**
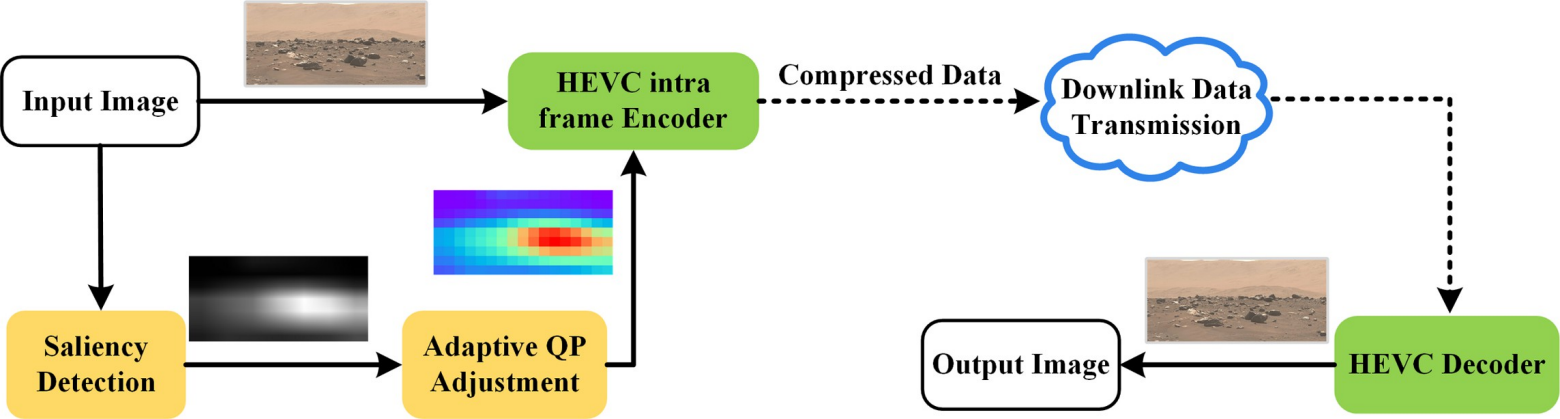
Flowchart of the proposed system. Onboard image compression comprises three stages: saliency detection by CNN generating a pixel-wise saliency map, CTU-level adaptive QP adjustment, and HEVC intra-coding.

In this work, to facilitate the planetary image compression process using a visual saliency-guided perceptual technique, a perceptual adaptive quantization method based on CNN and HEVC intra-coding has been proposed. To the best of our knowledge, this is the first effort toward developing a perceptual adaptive quantification technique to boost the performance of planetary image compression. The flowchart of the algorithm is represented in Fig 1. The experimental results prove that the proposed method can significantly improve the perceptual coding performance as compared to the standard HEVC coding.

The rest of this paper is organized as follows: Section 2 elaborates the performance analysis of the HEVC encoder and presents an overview of visual saliency detection. Section 3 introduces the proposed saliency detection method based on residual neural network. Section 4 represents a detailed description of the proposed adaptive perceptual quantification method. The performance analysis is assessed in Section 5. Eventually, Section 6 concludes this paper.

## 2. Related work

### 2.1 High efficiency video coding

HEVC is a new generation video compression standard after H.264/AVC [18]. The HEVC coding framework is extended through the adoption of advanced coding techniques. It is a comprehensive block-based hybrid compression scheme that provides multiple adjustable patterns and significantly improves the coding performance. The primary features include flexible recursive quadtree structure for block structure partitioning, multiple intra-prediction modes, Syntax-Based Context-Adaptive Binary Arithmetic Coding (SBAC) and Sample-Adaptive Offset (SAO) filtering. Although it has a design objective of better video compression, the Main Still Picture (MSP) profile of HEVC can be efficiently utilized to compress still images configured with the intra-coding pattern.

**Fig 2:**
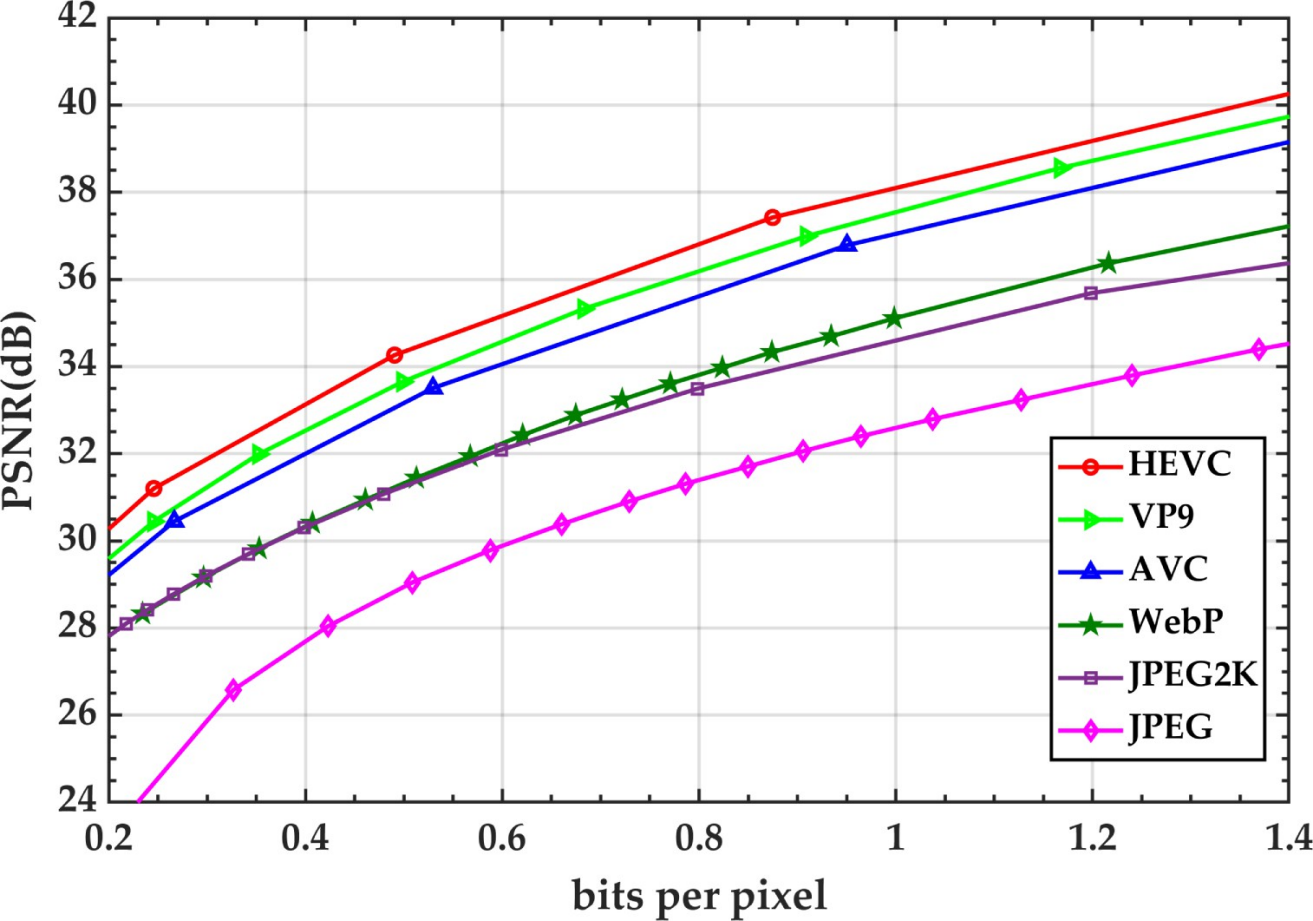
Rate-distortion performance of HEVC, JPEG, JPEG2000, WebP, VP9, and AVC codec over Kodak dataset. Kodak datasets consist of 25 lossless, true color (24 bits per pixel) PNG images of 768 × 512 pixels.

In this paper, the coding performance of HEVC is compared with the widely-applied image compression standards, including JPEG, JPEG2000, WebP [19], VP9 [20], and AVC based on Kodak [21] dataset. The Rate-Distortion (RD) curves of all the compression schemes are illustrated in Fig 2. As an overall observation, the results depict that the performance of HEVC is noteworthy. Accordingly, it is feasible to adopt the predictive coding approach of HEVC for high-resolution planetary images compression. The proposed work chiefly focuses on the improvement of HEVC intra-coding.

### 2.2 Visual saliency detection

Visual saliency is used to describe the prominent qualities of an object or region in a scene different from its surrounding neighborhood that attracts visual attention. Research shows that the Human Visual System (HVS) optimizes the allocation of visual information processing resources based on visual saliency analysis guided by the focus of attention mechanism, which corresponds to the highly discriminative and selective behavior displayed in visual neuronal processing [22].

Saliency detection can be defined as an automatic estimation of salient objects or regions of images without any prior assumption or knowledge [23]. The saliency map is a gray-scale image of the same size as the original image in which the value of each coordinate position reflects the saliency degree of the corresponding pixel in the image. In practice, saliency detection methods are utilized as the first step of many computer vision tasks. Being able to automatically, efficiently, and accurately identify the salient object regions can be helpful to allocate the finite computational resources for subsequent image processing, thereby improving the efficiency of information processing.

From the perspective of information processing mechanism, saliency detection approaches can be primarily categorized into two types: top-down models guided by subjective tasks and bottom-up models driven by objective content [24]. The top-down models are principally task-driven based on the semantic features to describe the specific objects and tasks determined by prior knowledge. They require abundant training data with human-labeled ground truth for training [25].

The bottom-up models are based on low-level visual features to compute saliency through the feature contrast between adjacent pixels or regions. Itti et al. [26] proposed an ITTI algorithm based on the visual perceptual field model of the biological neurocognitive theory. This model constructs a Gaussian pyramid with three feature channels of chromaticity, luminance, and orientation to compute the saliency maps across different scales. Harel et al. [27] introduced a novel graph-based normalization and combination strategy named GBVS. In this method, activation maps are formed on certain featured channels, which are further normalized such that they highlight conspicuously and admit the combination with other maps to obtain saliency map results. These two algorithms only consider the local feature comparison. LC [28], AC [29], and HC [30] algorithms use global statistical model features like color histograms with lower computational complexity. SR [31] and FT [32] algorithms are based on frequency domain analysis. CA [33] algorithm is based on contextual information that combines local and global feature contrast. DCLC [34] algorithm is based on prior compactness and local feature comparison fused with graph diffusion information.

## 3. Saliency map extraction

The objective of the perceptual-based image compression method is to improve the coding quality of the salient regions. Most scientists require a high-quality image with rich textured regions, which would be beneficial science targets in actual planetary exploration [35].Hence, the salient region detection is primarily focused here with no need to accurately detail the tight bound of the salient object in this paper.

With the incredible performance promotion of computer processors and the emergence of large-scale image datasets, deep learning (especially CNN) has attracted much attention due to its ability to extract high-level semantic information. It has shown commendable results in many datasets [36]. Unlike many traditional algorithms that depend on various forms of middle-level and low-level feature contrast or prior knowledge-based modeling, deep neural networks simulate the way of processing external information like HVS and form more abstract and robust high-level features. It does this by combining the underlying features which have stronger semantic perception characteristics, thereby exhibiting better generalization ability in practical applications.

**Fig 3:**
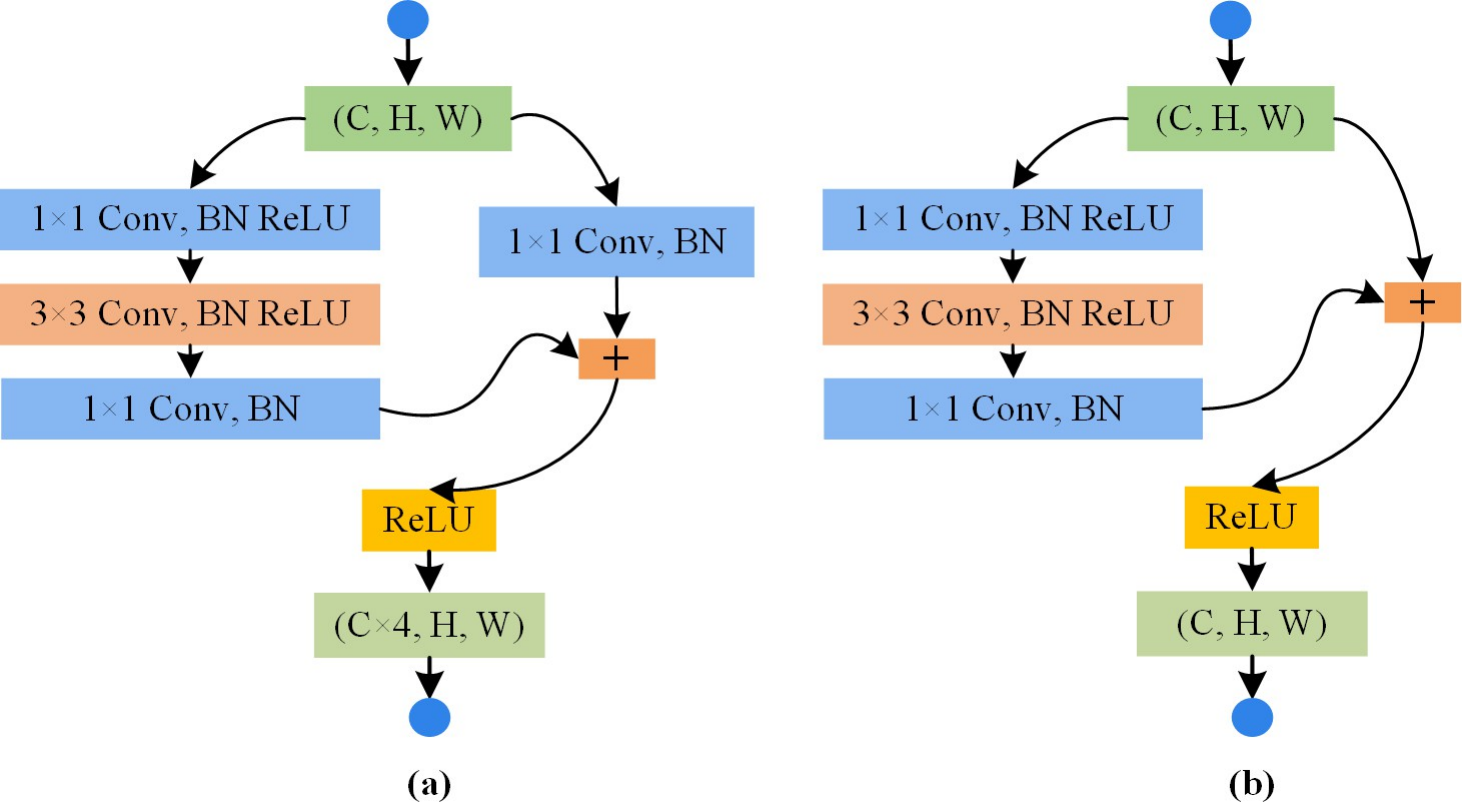
Two types of building blocks in ResNet50: (a) convolutional block; (b) identity block.

**Fig 4:**
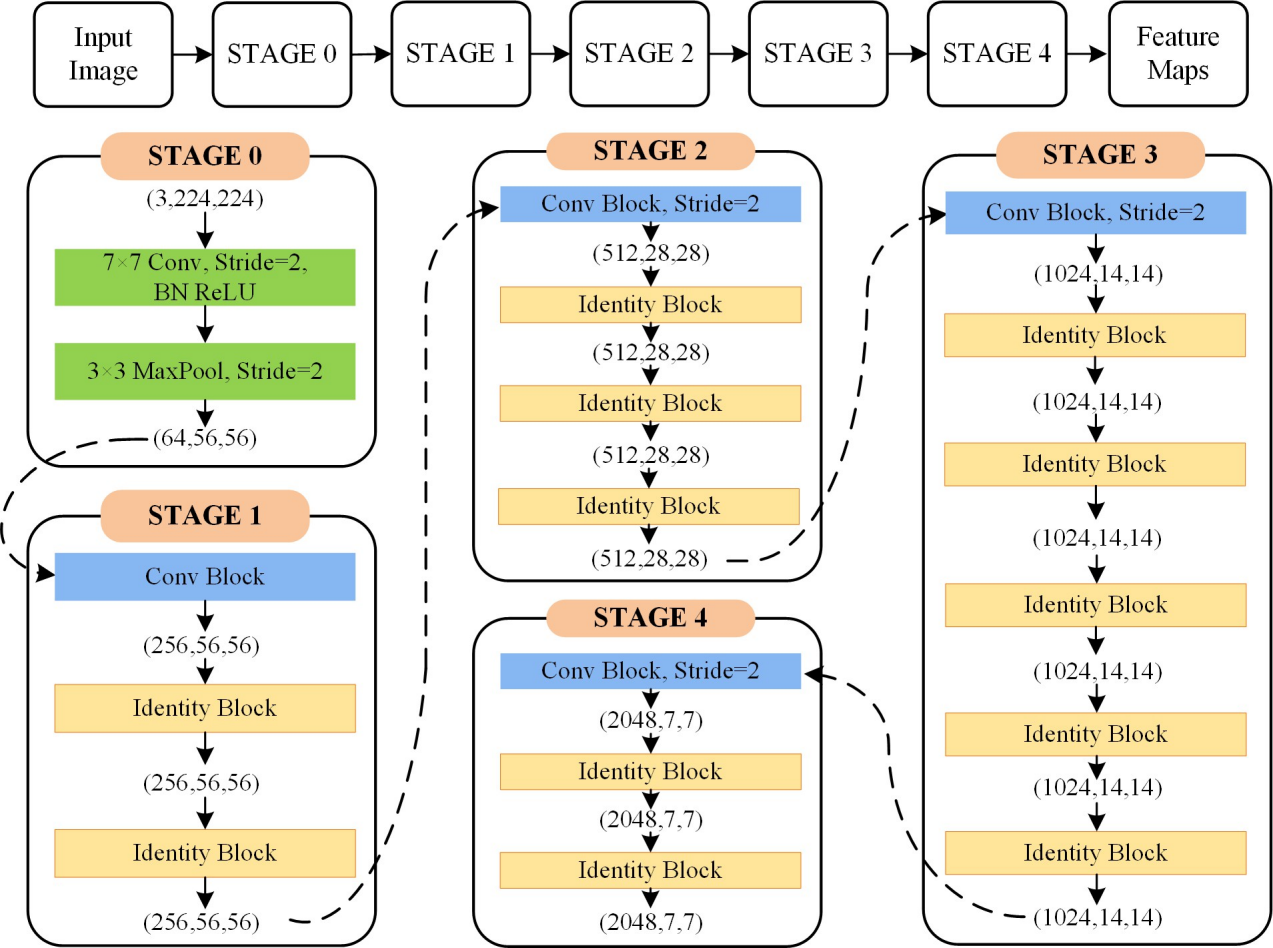
The architecture of deep convolutional layers in ResNet50.

**Fig 5:**
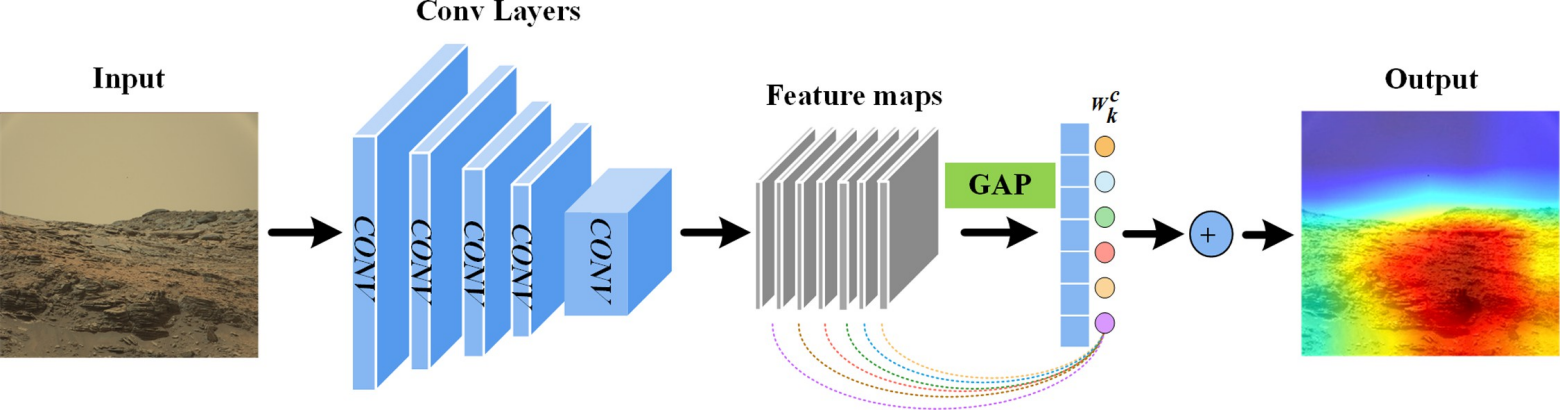
Architecture of proposed neural network for salient region detection.

The proposed salient region extraction network is developed on the basis of the deep residual network, i.e., ResNet50 [37]. Residual blocks with shortcut connections that perform identity mappings were introduced to solve the problem of overfitting. As depicted in Fig 3, two types of blocks are used in ResNet50: convolutional block in panel (a) and identity block in panel (b). A convolutional block is almost similar to the identity block but there is a convolutional layer in a short-cut path to alter the dimension such that the dimension of input matches the dimension of output. Identity Block is used when there is no difference in the input and output dimensions. ResNet50 is a multi-layer neural network with a supervised learning architecture made up of two parts: a feature extractor and a trainable classifier. Cascaded convolutional filters can be assessed as a local feature extractor to identify the relationship between the pixels and produce high-level feature maps. The architecture of deep convolutional layers in Resnet50 is highlighted in Fig 4. Since it was originally developed for the image classification task, the structure has been modified here to develop salient region localization by exploiting repeated convolutional layers and the Global Average Pooling (GAP) layer, as illustrated in Fig 5.

Inspired by the Class Activation Mapping (CAM) proposed in [38] and Gradient-weighted CAM (Grad-CAM) in [39] to identify the discriminative regions based on CNNs, the output of the last convolutional layer of Resnet50 is used as high-level feature representation. The CNN features are concatenated with the GAP layer. GAP is performed on the convolutional channel feature maps, and its output is the spatial average of each feature map. The resulting vector is further fed into the SoftMax layer, which outputs the predicted class. Each node in the GAP corresponds to a specific feature map and the corresponding link weight between the GAP layer and the SoftMax layer highlights the importance of the predicted class. The class activation map is obtained by a linear weighted sum of the feature maps. For a given class ***c***, the linear fusion pattern is based on the following Eq 1.

$$S^{c}\leftarrow \text{ReLu}\left(\sum_{k}w_{k}^{c}A^{k}\right)$$ (1)

where ReLU indicates the linear rectified function that retains the input greater than 0 and eliminates the negative influence of less probable classes, **A**^*k*^represents the *k*_*th*_feature map, and the weight $w_{k}^{c}$ indicates the importance of **A**^*k*^ for class *c*.

In this paper, instead of identifying a particular class, the confidence scores and the output tensor from the SoftMax layer are ranked to obtain the top-five highest class elements *c* = {*c*_*i*_}, *i* = 1,2,3,4,5. Their linear weighted fusion feature maps are further calculated and merged. Finally, the fused feature map is resized and interpolated according to the size of the original input image to obtain the final output saliency map result as shown in Eq 2.

$$Sal\leftarrow Upsample\left(\sum_{i}S^{c_{i}}\right)$$ (2)

Based on the proposed method, we can only take one step of forward-pass inference to extract the saliency map. To optimize the generalization ability of the residual neural network and reduce training time, the pre-trained ResNet50 model is utilized in this study. After the process of pre-training, the neural network has been endowed with certain understanding ability of image information, which is in forms of the weight parameters within the neural network. We remove each convolution layer from back to front and then concatenated GAP layer and SoftMax layer in turn to explore which output could be the best feature representation. And each time we would freeze weights of convolution layers and only retrain the SoftMax layer on ImageNet from the Large Scale Visual Recognition Challenge 2012 (ILSVRC2012) [40] based on the transfer learning strategy. Finally, we exploit the output of the last convolutional layer as high-level feature representation of images to obtain salient information.

## 4. Perceptual adaptive quantification

The QP related to the quantization step in the encoding process directly affects the bits assigned to each CTU and the ultimate image visual quality. In HEVC encoding, each frame is partitioned into Coding Tree Units (CTUs) of 64 × 64 samples. This paper proposes a CTU level QP setting technique that utilizes the visual saliency information to adjust the quantization parameter adaptively. Based on the saliency map obtained in the preceding section, every CTU is assigned a QP offset (Δ*QP*) according to the saliency degree. The final QP value of every CTU is *QP*_*init*_ + Δ*QP*, where *QP*_*init*_ is the base QP assigned initially.

The proposed adaptive quantification parameter for a CTU is determined by two primary factors and calculated as mentioned in Eq 3.

$$\Delta QP=QP_{weighted}+QP_{sal\;\_\;offset}-QP_{init}$$ (3)

Where *QP*_*init*_ denotes the initial frame-level quantization parameter and Δ*QP* maps the difference of the QP in current CTU with respect to *QP*_*init*_, *QP*_*weighted*_ represents the global saliency contrast-driven weighted quantization component for a CTU, and *QP*_*sal_offset*_ indicates the perceptual quantization component based on local saliency perception.

The weighted quantization component *QP*_*weighted*_ is given by:

$$QP_{weighted}=round\left(\frac{QP_{init}}{\omega }\right)$$ (4)

where the weights are obtained by global saliency contrast as given in the following Eq 5:

$$\omega =\sqrt{\frac{Sal_{cu\_avg}}{Sal_{\text{avg}}}}$$ (5)

*Sal*_*cu*_*avg*_ and *Sal*_*avg*_ are the mean of the corresponding saliency map for the current CTU and the whole image, respectively.

The perceptual quantization component *QP*_*sal_offset*_ is obtained from Eq 6. The overall saliency of each CTU is logarithmically calculated to determine *QP*_*sal_offset*_.

$$QP_{sal\_offset}=clip\left(\beta \log_{2}Sal_{cu_{-}sum},-3,-3\right)$$ (6)

Where *Sal*_*cu*_*sum*_ indicates the total value of the saliency map for the current CTU. *β* is defined empirically and set to 0.3. The function of *clip* is to limit the output *QP*_*sal_offset*_ value between the first parameter (minimum value) and the second parameter (maximum value).

**Fig 6:**
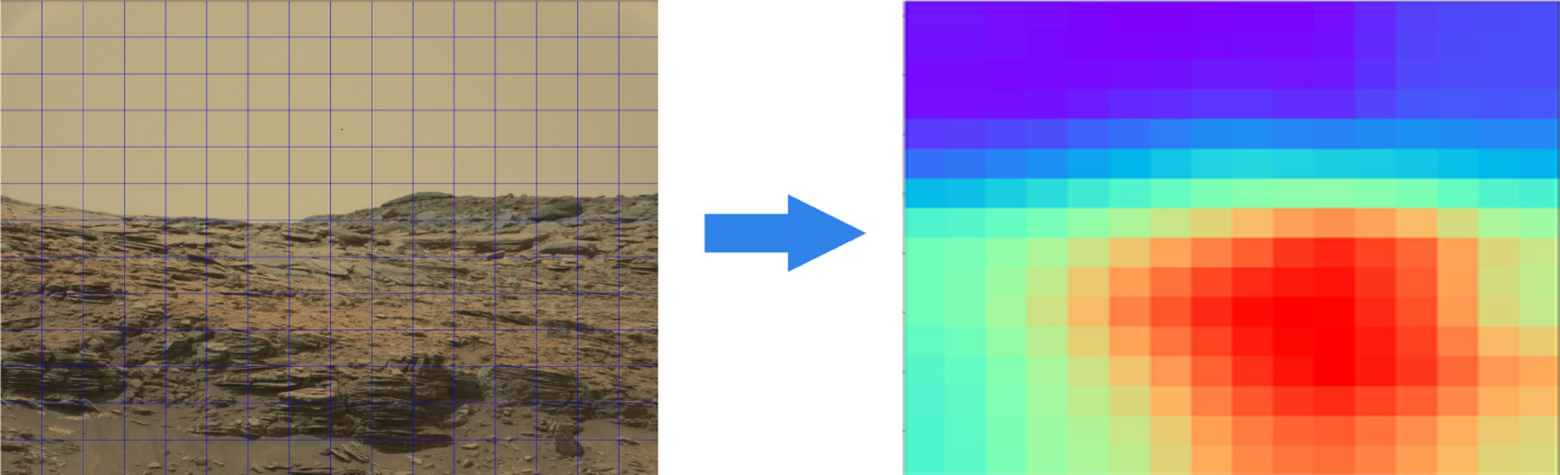
QP distribution map.

Through the aforementioned process, the QP offset values of all the CTUs forming a non-uniform delta quantization parameter map as a function of the ROI across the image are determined. This is highlighted in Fig 6. On the left of the panel is an example of the partition of the original image according to the CTU size. The colors of the corresponding blocks in the right panel indicate the QP offsets computed by the proposed algorithm according to the saliency. The salient regions will be assigned smaller QPs to reduce the loss in image quality and coarse quantization will be adopted to the non-salient regions for overall bitrate savings.

## 5. Experimental results

This section evaluates the performance of the proposed saliency model and compares it with several conventional models and state-of-the-art CNNs. Further, the coding performance of the proposed method compared with the HEVC is presented. The established test image dataset and the specific experimental details are elaborated, and the corresponding results are mentioned. This section highlights the qualitative and quantitative results as well as the corresponding analysis.

### 5.1 Source dataset

In the next Section 5.2, we conducted experiments to evaluate saliency mode performance on a small portion of the mars32k dataset (available at https://dominikschmidt.xyz/mars32k/). This dataset is an unlabeled public dataset that contains 32,000 color images collected by the Curiosity rover on Mars between August 2012 and November 2018. The images show various geographical and geological features of Mars such as mountains and valleys, craters, dunes and rocky terrain. In the Section 5.3, 50 raw Curiosity images with a high resolution of 1344 × 1200 provided by NASA/JPL (available at https://mars.nasa.gov/msl/multimedia/raw-images) are used to verify the coding performance. Note that the proposed method allows the input image to be of arbitrary size and resolution.

### 5.2 Saliency mode performance

#### 5.2.1 Implementation details

**Fig 7:**
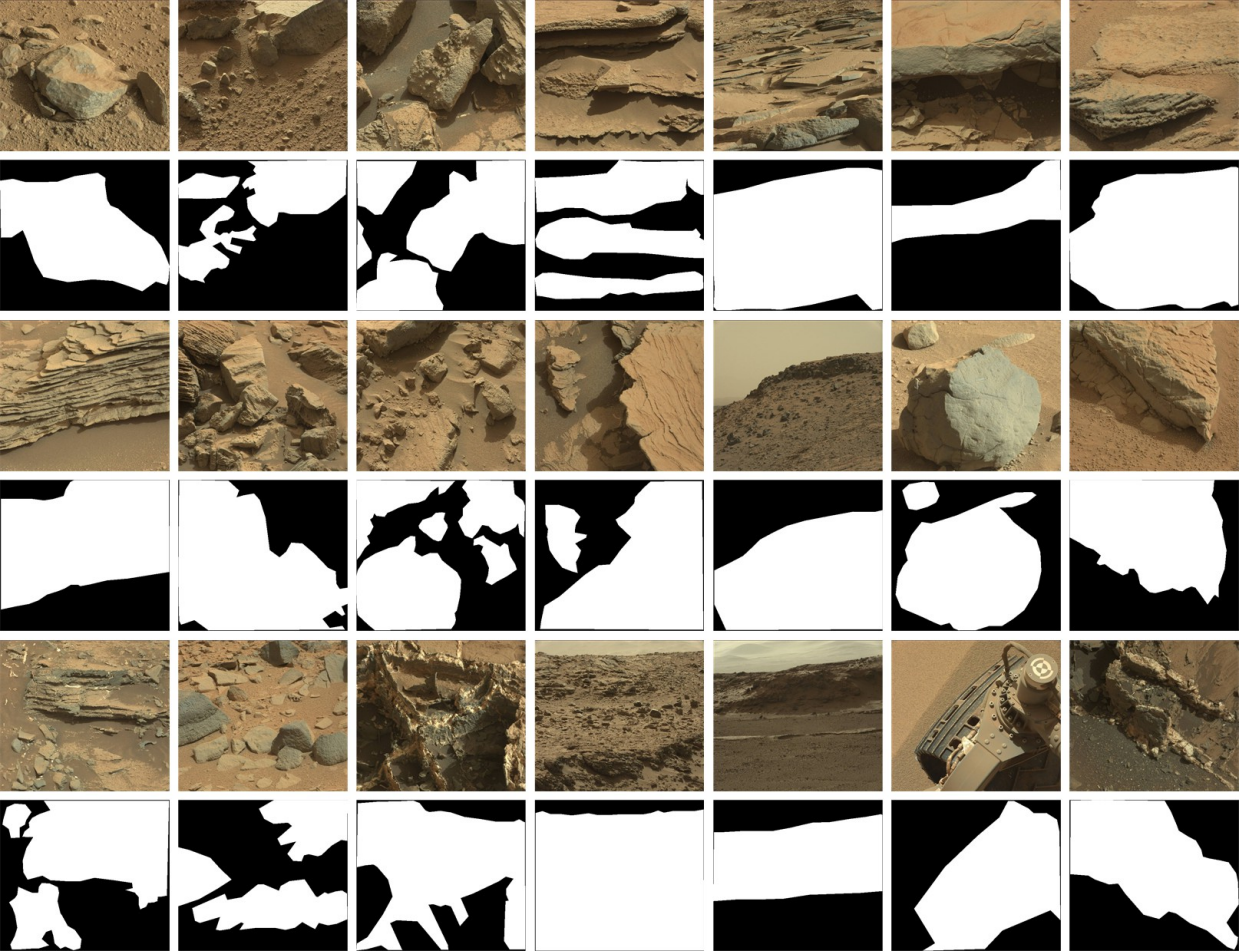
Examples of saliency maps over the test images: Original images and corresponding saliency map masks.

In order to quantitatively evaluate the saliency detection results, we have tried our best to label the pixels of salient objects or regions in 50 images based on what we know so far that particularly scientists’ interest in Mars. Fig 7 depicts some test images and the corresponding saliency masks. When we build the ground-truth of salient objects or regions, we adhere to the following rules: 1) the generation of candidate salient objects doesn’t rely on category specific information; 2) we preferentially label rocks, debris or something with definite shaped boundaries in Mars; 3) the whole region with abundant texture features will be marked when there is no prominent object.

We adopt four widely used evaluation metrics on saliency object detection [41] to evaluate all methods comprehensively: Structure-measure (*S*_*m*_) [42], Maximum F-measure (maxF), Maximum enhanced-alignment measure ($E_{\xi }^{\max}$) [43] and Mean Absolute Error (MAE). *S*_*m*_ evaluates region-aware and object-aware structural similarity and maxF jointly considers precision and recall under the optimal threshold. $E_{\xi }^{\max}$ simultaneously considers pixel-level errors and image-level errors. MAE computes pixel-wise average absolute error. Additionally, to fully evaluate the actual efficiency of the saliency detection technique, we test the practical computing time of every method on a PC with a 3.40 GHz Intel Core i7-6700 Processor. And we report the total number of network parameters (Params) and theoretical amount of floating point arithmetics (FLOPs) to analyze the complexity of all the network architectures.

#### 5.2.2 Performance comparison

Planetary images are characterized by unknown semantics but rich textures. Under these circumstances, it is tough to translate the prior knowledge into ROI. Hence, the saliency detection technique can only be based on the actual information contained in an image. Thereupon, the proposed saliency model is compared with 9 bottom-up saliency methods described in Section 2.2 to prove the effectiveness.

Beyond the comparison with classic saliency detection algorithms, we also compare the saliency maps generated by different network architectures to verify the accuracy. We compare the proposed method with 4 state-of-the-art networks including SKNet [44], ResNeXt [45], Res2Net [46] and ResNeSt [47]. Furthermore, considering that typical onboard remote sensing systems have limited storage and compute capacity, we compare the proposed model with another 4 state-of-the-art lightweight CNN architectures including ShuffleNet_v2 [48], MobileNet_v3 [49], EfficientNet_b0 [50] and GhostNet [51]. The experimental results on the test image dataset are given in Tables 1 and 2.

**Table 1:** Quantitative comparison of our proposed modified residual architecture with 9 traditional bottom-up saliency methods. Text in bold denotes the best results.

Model	*S*_*m*_ ↑	maxF ↑	$E_{\xi }^{\max}↑$	MAE ↓	Time(s)
ResNet-50	**0.4536**	0.7311	**0.4280**	**0.4480**	2.10
ITTI	0.4314	**0.7714**	0.4258	0.4829	2.81
GBVS	0.4248	0.7460	0.4276	0.4959	0.55
LC	0.2386	0.6928	0.3024	0.5947	1.26
AC	0.2506	0.6937	0.3019	0.5816	0.78
HC	0.2160	0.6928	0.2657	0.6145	5.12
SR	0.2546	0.6928	0.3022	0.5900	0.68
FT	0.2475	0.6928	0.2979	0.5897	0.79
CA	0.3853	0.7512	0.4042	0.5111	41.43
DCLC	0.3764	0.7620	0.4187	0.5089	4.89

**Table 2:** Quantitative comparison of our proposed modified residual architecture with 8 state-of-the-art methods for saliency map extraction. The best and the second-best results are highlighted in bold.

Model	*S*_*m*_ ↑	maxF ↑	$E_{\xi }^{\max}↑$	MAE ↓	Time(s)	FLOPs(G)	Params(M)
ResNet-50	0.4536	**0.7311**	**0.4280**	**0.4480**	2.10	4.12	25.6
SKNet-50	**0.4768**	0.7136	0.4274	**0.4423**	2.88	4.51	27.5
ResNeXt-50	**0.4732**	0.7151	**0.4280**	0.4498	2.22	4.27	25
Res2Net-50	0.4442	**0.7232**	**0.4429**	0.4618	2.29	4.29	25.7
ResNeSt-50	0.3630	0.7041	0.3672	0.4925	5.13	5.41	27.5
ShuffleNetv2	0.4452	0.7254	0.4126	0.4801	0.62	0.60	2.3
EfficientNet-b0	0.3728	0.7115	0.3472	0.4787	1.17	0.39	5.3
GhostNet_1x	0.4148	0.7148	0.3931	0.4704	0.97	0.14	5.18
MobileNetv3_large	0.3450	0.7093	0.3567	0.5175	0.78	0.22	5.48

The quantitative comparison results show that the modified ResNet-50 outperforms the other models on the test image dataset, with comparable number of parameters and FLOPs, hence demonstrating the great effectiveness of the proposed method. It achieves a compromise between saliency detection performance and computational complexity, which can better adapt to dynamic requirements.

**Fig 8:**
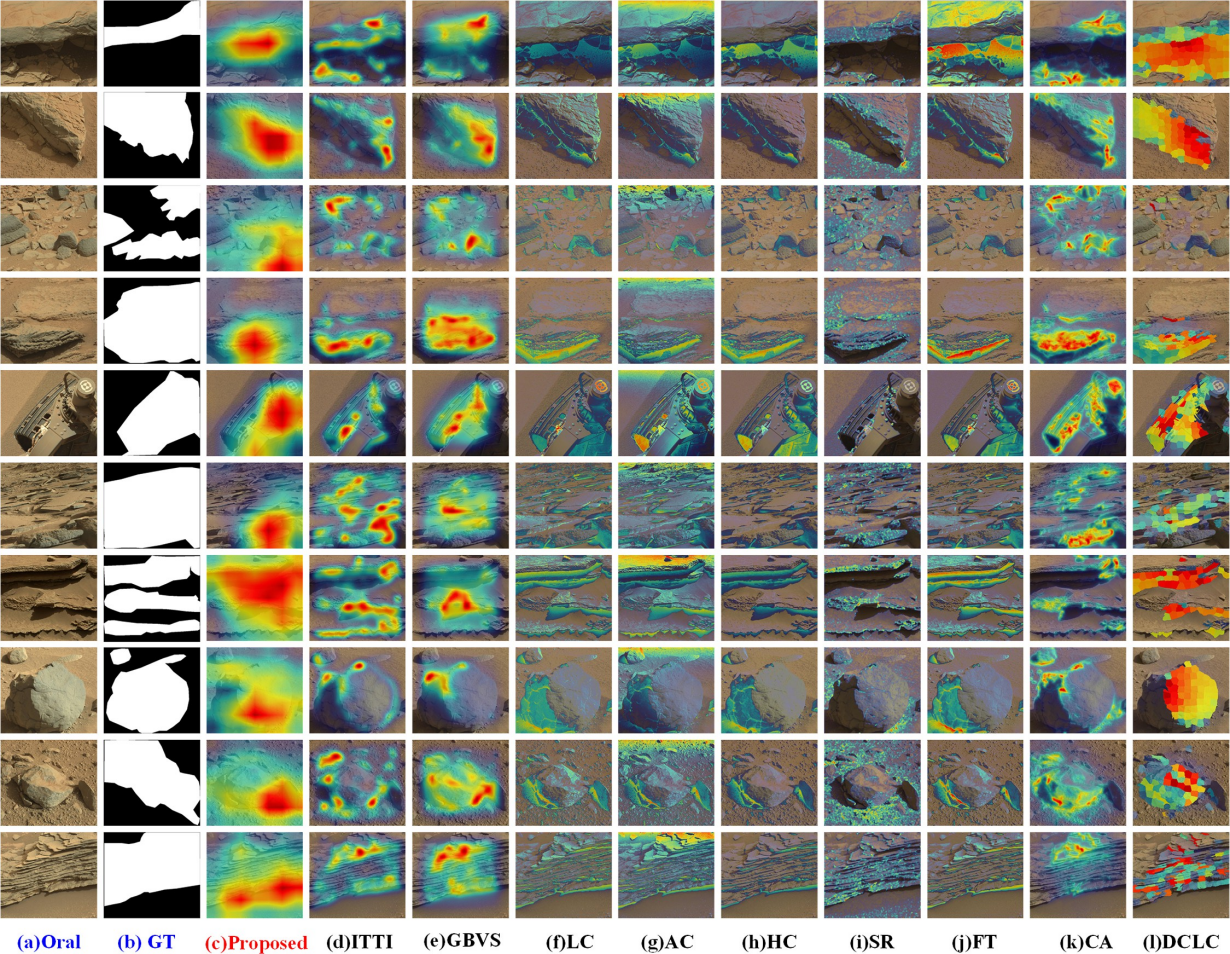
Performance comparison with eight classic bottom-up saliency methods.

**Fig 9:**
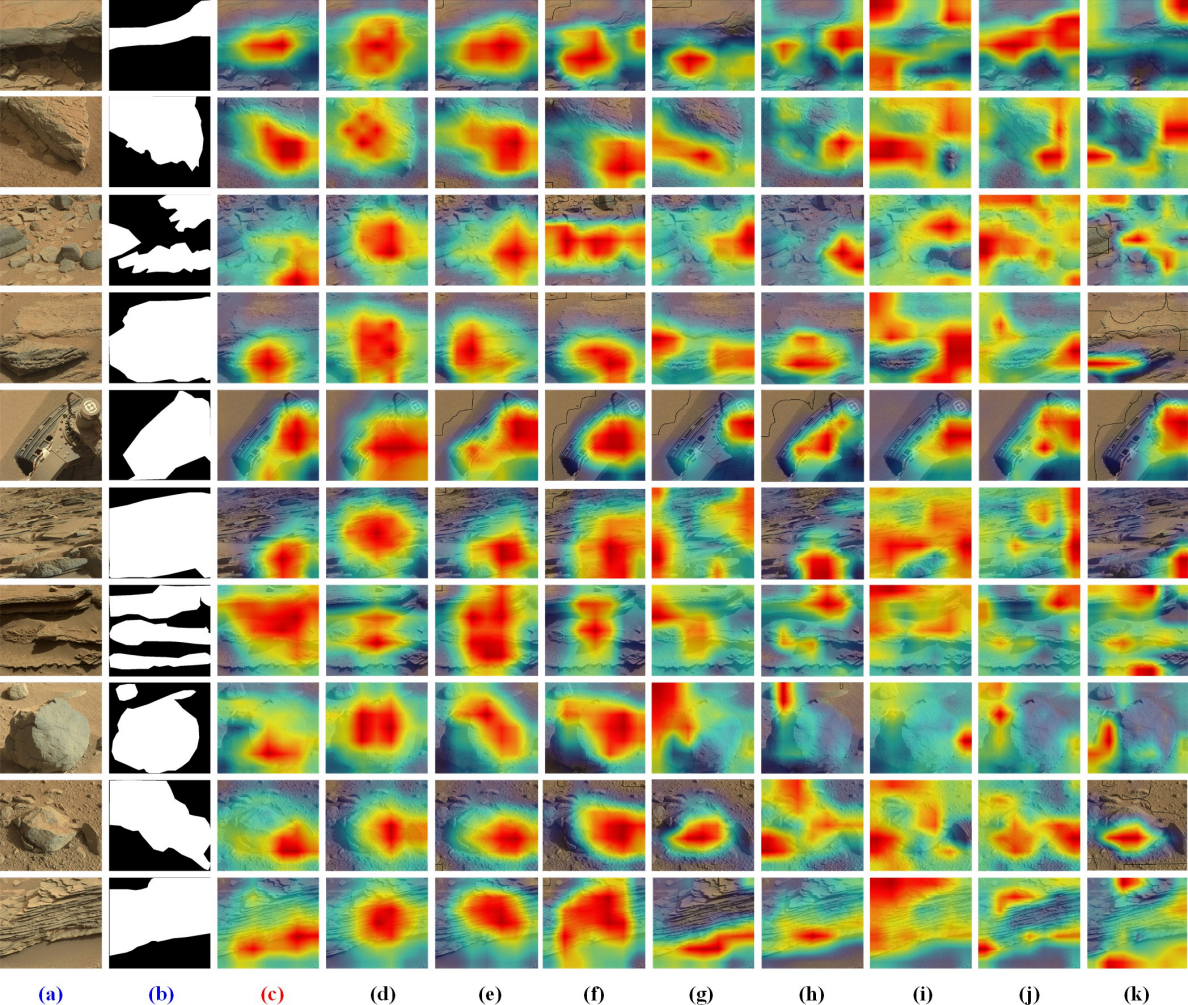
Performance comparison of the proposed method with the latest state-of-the-art methods. (left to right: original image, Ground Truth, ResNet, SKNet, ResNeXt, Res2Net, ResNeSt, ShuffleNet_v2, MobileNet_v3, EfficientNet_b0, and GhostNet).

We also show visual comparison results of our method with respect to others. The results are visually represented and displayed in the form of a heatmap generated by overlaying the saliency map onto the original image, as highlighted in Figs 8 and 9. In the heatmap results, brighter regions indicate the salient locations to which a human observer pays more attention, while the darker areas represent the less-saliency regions.

Based on the purpose of semantic perceptual compression, the extracted saliency map is expected to cover as many significant objects as possible without a precise semantic segmentation. There are many rocks, gravel, and other unknown targets with rich textures and diverse sizes but single morphology on the surface of Mars. According to the experimental results, the classic saliency models cannot cover all the salient objects or regions while being more sensitive to the intense edges. However, the proposed algorithm based on CNN can detect the salient regions with better accuracy as visible. Furthermore, the results suggest that the deep layer features in CNN are the texture-biased representation. The robustness and generalization of deep features make it effective to apply transfer learning for planetary image processing in the challenging scenario of Mars’ surface where exists salient objects in all shapes and sizes, foreground and background having similar appearances, and cluttered backgrounds.

### 5.3 Perceptual coding performance analysis

#### 5.3.1 Implementation setup

The hardware configuration for experimental validation is as follows: Intel i7–6700CPU @3.40 GHz with 36 GB RAM, 64-bit Microsoft Windows 10, and the fastest practical academic open-source solution Kvazaar v2.0.0 [52] for HEVC intra-coding. The source code of the Kvazaar project is publicly available and can be accessed directly from GitHub (https://github.com/ultravideo/kvazaar). The encoder is configured to the intra-only operation mode, which is also implied by the MSP profile constraints.

Image quality assessment is the quantification of human perception of image quality. In this paper, considering the actual perceived visual quality, the following measure metrics aligned with the human visual system are exploited to reach a more accurate assessment of visual quality for the sake of verifying the coding performance.

Peak Signal-to-Noise Ratio (PSNR) is a commonly used objective visual quality assessment metric. It is a pixel-level metric obtained by calculating the difference between the corresponding pixel points of two images. Only by some mathematical calculations, PSNR cannot reflect the actual visual perceptual quality since there is no consideration of the human visual characteristics like visual acuity and visual masking effects.

PSNR-HVS [53] and PSNR-HVS-M [54] are advanced versions of PSNR associated with HVS. PSNR-HVS integrates the results of error sensitivity, structural distortion, and edge distortion. PSNR-HVS-M considers the contrast sensitivity function and contrast masking of DCT domain-based boundary limiting mechanism to achieve the overall perceptual optimization.

Structural Similarity Index (SSIM) [55] is another perceptual metric determined by the comparison of luminance, contrast, and structure information to measure structural similarity. The Multi-Scale SSIM (MS-SSIM) [56] is an extension of SSIM incorporating the variations of viewing conditions.

Visual Information Fidelity at Pixel Domain (VIFP) [57] measures the quality of the image based on the mutual information between the reference image and the image to be evaluated.

Furthermore, the saved coding bitrates are calculated by the following Eq 7, where *R*_*std*_ and *R*_*pro*_ denote the coding bits obtained by the standard HEVC and proposed perceptual method respectively.


$$\Delta BitrateSaving=\frac{R_{std}\text{-}R_{pro}\;}{R_{std}}\times 100\%$$ (7)

#### 5.3.2 Objective image quality evaluation

The configuration has been modified to encode with QPs of {22, 25, 28, 32, 35, 38, 41, 44, 47, 51} and obtain ten groups of comparison results. The evaluation is performed by keeping the same values of initial QP for all the images and calculating the mean PSNR, PSNR-HVS, PSNR-HVS-M, SSIM, MS-SSIM, and VIFP.

The results are enlisted in Table 3. It can be observed that the average coding bit rate decreases by 4.20% as compared to the standard HEVC, and the largest reduction is 7.17% accompanied by the stability and optimization of image quality. The proposed approach enhances the PSNR, PSNR-HVS, and PSNR-HVS-M by 0.69 dB, 0.77 dB, and 1.07dB, respectively. As for SSIM, MS-SSIM, and VIFP, this method also achieves higher scores for all the test images. It thus justifies the effectiveness of the proposed perceptual image compression algorithm.

**Table 3:** Performance metrics of proposed algorithm versus the standard HEVC codec under different initial QPs.

QP	ΔBitrateSaving (%)	ΔPSNR(dB)	ΔPSNR-HVS (dB)	ΔPSNR-HVS-M (dB)	ΔSSIM	ΔMS-SSIM	ΔVIFP
22	2.239	-0.1917	-0.2160	-0.4028	-0.0004	0.0001	-0.0041
25	3.604	0.0789	0.0046	-0.1600	-0.0003	0.0001	0.0016
28	1.080	0.2911	0.2903	0.3315	0.0001	0.0001	0.0069
32	1.704	0.4535	0.5041	0.6961	0.0000	0.0001	0.0110
35	5.524	0.6411	0.7386	1.0625	0.0008	0.0001	0.0170
38	6.973	0.7874	0.9150	1.3346	0.0015	0.0003	0.0220
41	5.116	0.9502	1.1022	1.6119	0.0031	0.0014	0.0293
44	4.119	1.1017	1.2800	1.8451	0.0051	0.0026	0.0354
47	4.503	1.2306	1.4085	2.0093	0.0075	0.0046	0.0396
51	7.171	1.5126	1.7135	2.3932	0.0178	0.0142	0.0528
**Avg**	**4.203**	**0.6855**	**0.7741**	**1.0721**	**0.0035**	**0.0024**	**0.0212**

**Fig 10:**
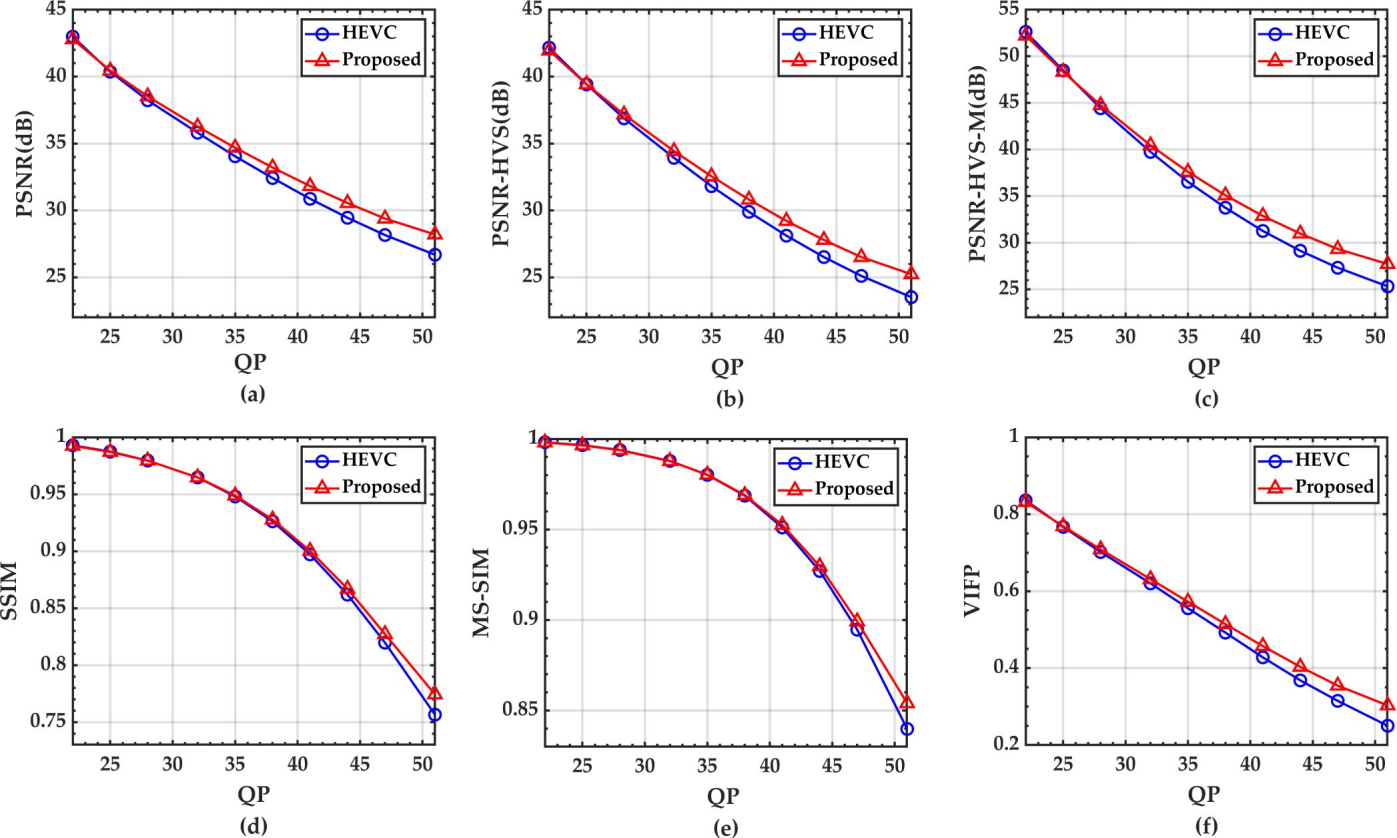
RD curves for the proposed algorithm and the standard HEVC codec for 50 images of the planet Mars under different initial QPs: (a)PSNR (b)PSNR-HVS (c)PSNR-HVS-M (d)SSIM (e)MS-SSIM (f)VIFP.

To describe the coding performance intuitively, Fig 10 illustrates the RD curves where the rates are in bits-per-pixel vs. the value of distortion functions based on the previously measured metrics. The proposed method is indeed capable of preserving the visual quality without sacrificing coding performance. All the image quality evaluation metrics have noticeably improved by this method.

#### 5.3.3 Subjective image quality assessment

**Fig 11:**
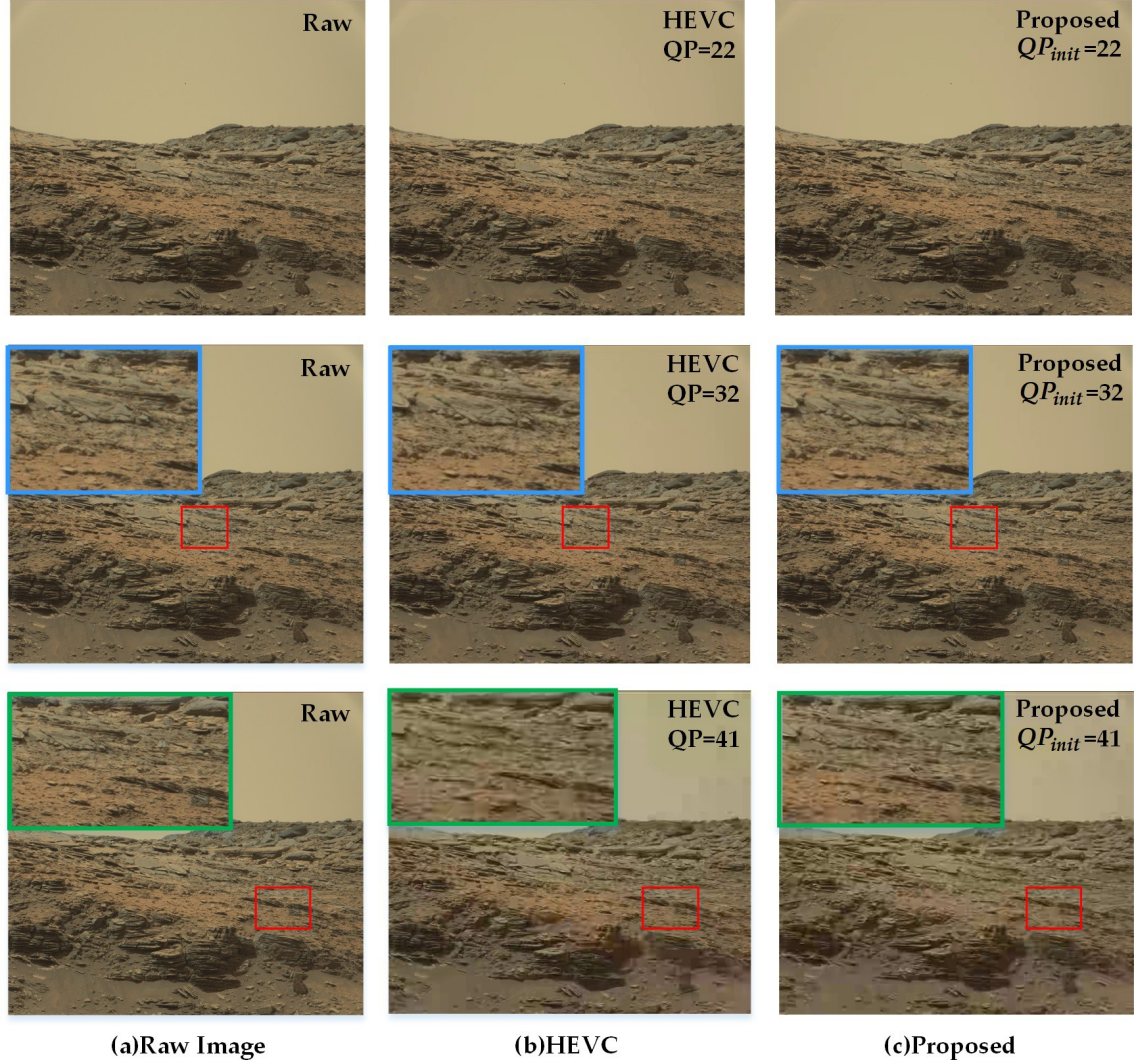
Subjective quality comparison between the standard HEVC codec and the proposed adaptive quantization algorithm at different QP setting. Details are magnified by bilinear interpolation for comparison.

**Fig 12:**
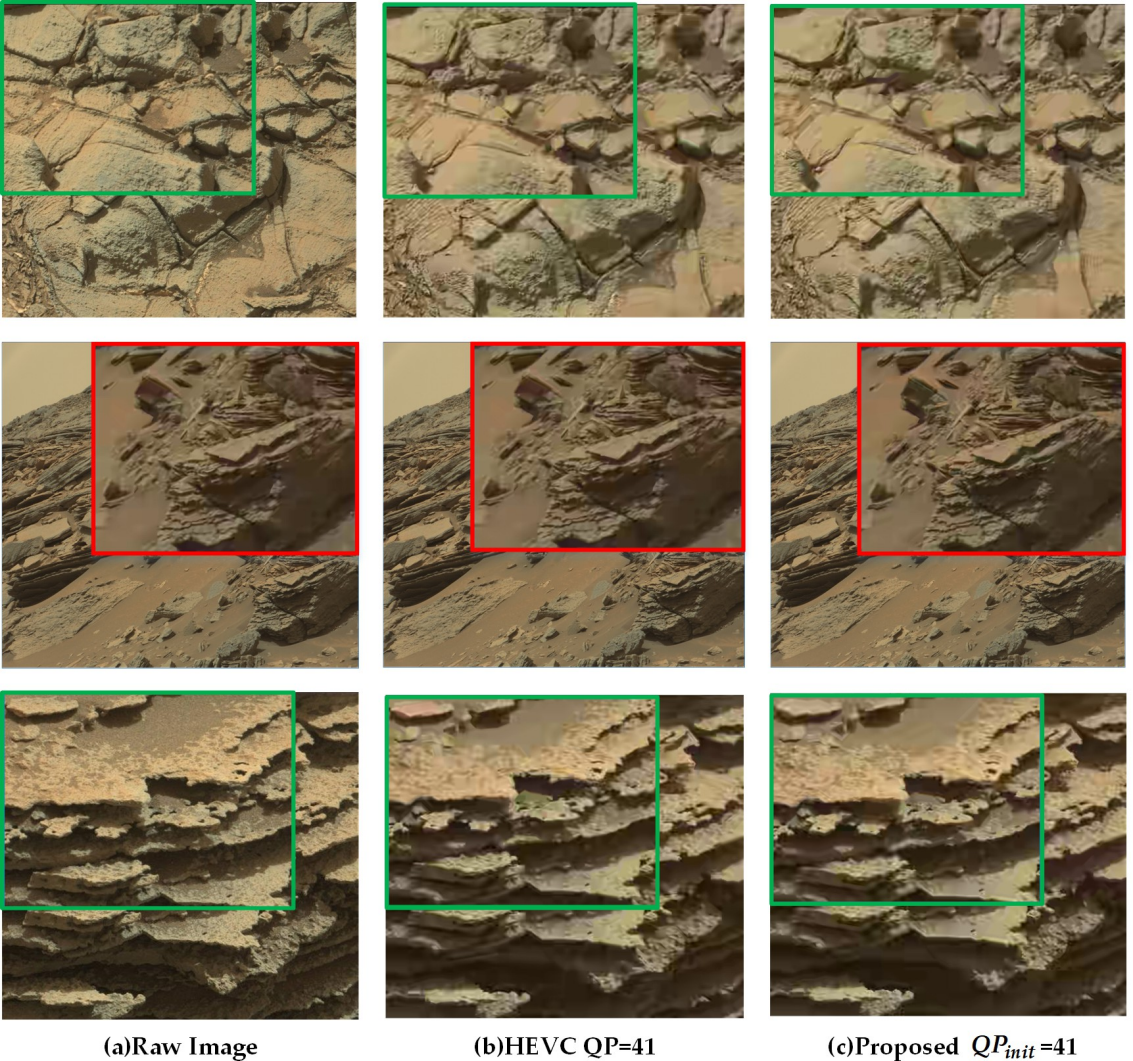
The figure displays qualitative results comparison at three groups of images when QP is set to 41. For ease of comparison, details are magnified by bilinear interpolation.

For qualitative analysis, several groups of reconstructed images displaying the visual quality are represented in Figs 11 and 12. Fig 11 shows one of the original test images in panel (a). The images encoded by the standard HEVC and proposed method under QP = 22, 32, 41 can be seen in panels (b) and (c).

On the whole, the compressibility increases, and the image quality degrades with the increase in QP. As illustrated in Fig 11, as compared to the original image, both the encoded images hardly have any subjective image quality loss at QP = 22. At QP = 32, the quality loss of both the images is still acceptable. While at QP = 41, the compression ratio gets high, blocking effect appears in images, and the proposed method captures more fine-grained details in the salient areas with rich texture features. This suggests that the current research produces a better effect for a high compression ratio. The distortion from compression in smooth regions with low-frequency features can be more acceptable for little influence over the scientific interpretation.

To further confirm the strength of the proposed method in low bitrate, Fig 12 demonstrates the subjective quality comparison results of three different images at QP = 41. As seen in the figure, the standard HEVC scheme exhibits significant performance degradation with blurring and color distortion. However, the proposed algorithm can still maintain the visual quality of the significant regions, and the compression fidelity is relatively better in the textured edges and parts of the images with rich details. Similar performance gain can also be acquired in other images.

## 6. Conclusions

In the present study, a visual saliency guided perceptual image compression method is suggested to achieve better subjective HEVC intra-coding performance for the planetary images. This is the first effort toward developing a perceptual adaptive quantification technique for onboard planetary image compression. The proposed algorithm utilizes a modified model based on pre-trained ResNet50 to extract the saliency map, which indicates the preference of texture features in CNN. With the help of the saliency map, the adaptive QP offsets are calculated for each CTU. In the proposed CTU-level QP adjustment technique based on global saliency contrast and local saliency perception, each CTU block is assigned to a different quantization parameter instead of the concept of the whole image using the same quantization parameter for bitrate saving. According to the saliency map, the salient region that matches the human visual perceptual properties will be encoded in fine quantization, while the flat regions encoded in coarse quantization. Experimental results on raw planetary images of Mars prove that the proposed system achieves 1.08% to 7.17% reduction in bitrate and preserves better visual quality compared to the HEVC anchor. The visual quality assessment verification based on multiple quantitative measure metrics that correlate with the visual perception mechanism comprehensively justifies the effectiveness of the proposed scheme in maintaining the visual quality of an image.
